# Determinants of survival in patients with brain metastases from cutaneous melanoma

**DOI:** 10.1038/sj.bjc.6605622

**Published:** 2010-04-06

**Authors:** M Staudt, K Lasithiotakis, U Leiter, F Meier, T Eigentler, M Bamberg, M Tatagiba, P Brossart, C Garbe

**Affiliations:** 1 Section of Dermatological Oncology, Department of Dermatology, Central Malignant Melanoma Registry of the German Dermatological Society, Eberhard-Karls-University, Tuebingen, Germany; 2 Department of Radio-Oncology, Eberhard-Karls-University, Tuebingen, Germany; 3 Department of Neurosurgery, Eberhard-Karls-University, Tuebingen, Germany; 4 Department of Haematology and Oncology, Rhenish Friedrich-Wilhelm University, Bonn, Germany

**Keywords:** brain metastasis, cutaneous melanoma, survival, stereotactic radiosurgery, neurosurgery, serum lactate dehydrogenase

## Abstract

**Background:**

This retrospective study aimed to identify prognostic factors in patients with brain metastases from cutaneous melanoma.

**Methods:**

In all, 265 patients under regular screening according to valid national surveillance guidelines were included in the study. Kaplan–Meier analyses were performed to estimate and to compare overall survival. Cox modeling was used to identify independent determinants of the overall survival, which were used in explorative classification and regression tree analysis to define meaningful prognostic groups.

**Results:**

In total, 55.5% of our patients presented with two or less brain metastases, 82.6% had concurrent extracranial metastasis and 64% were asymptomatic and diagnosed during surveillance scans. In all, 36.7% were candidates for local treatment (neurosurgery or stereotactic radiosurgery (SRS)). The median overall survival of the entire collective was 5.0 months (95% confidence interval: 4.3–5.7). Favourable independent prognostic factors were: normal pre-treatment level of serum lactate dehydrogenase (*P*<0.001), administered therapy (neurosurgery or SRS *vs* other, *P*=0.002), number of brain metastases (single *vs* multiple, *P*=0.032) and presence of bone metastasis (false *vs* true, *P*=0.044). Three prognostic groups with significantly different overall survival were identified. Candidates for local treatment (group I) had the longer median survival (9 months). Remaining patients could be further classified in two groups on the basis of serum lactate dehydrogenase.

**Conclusion:**

Applied treatment and serum lactate dehydrogenase levels were independent predictors of survival of patients with brain metastases from cutaneous melanoma. Patients receiving local therapy have overall survival comparable with general stage IV melanoma patients.

Cutaneous melanoma is one of the most common causes of cerebral metastasis (Zimm *et al*, 1981; Nussbaum *et al*, 1996). Large clinical series show that brain metastases are diagnosed in up to 10% of melanoma patients during their lifetime and autopsy data show that up to 73% of patients who die from disseminated cutaneous melanoma have brain involvement (de la Monte *et al*, 1983; Sampson *et al*, 1998). Cerebral metastasis is a very distressing event in the natural course of melanoma because it carries the worst prognosis of all visceral metastases and represents a major cause of death in patients with disseminated disease (Brand *et al*, 1997).

Current management of cerebral metastasis depends largely on the number and the size of lesions and on the extracranial extension of metastatic disease. It involves neurosurgery, stereotactic radiosurgery (SRS), whole brain radiation therapy (WBRT) and chemotherapy. Neurosurgery is usually offered to patients with single or a few metastatic lesions, provided that brain metastases are surgically accessible and that the patient can tolerate the operation. Stereotactic radiosurgery is offered as an alternative to direct neurosurgery in patients with a few brain metastases, which cannot be surgically removed and WBRT is used in conjuction with the neurosurgery or SRS because it is associated with better local control (Eichler and Loeffler, 2007). Such intervention can provide effective palliation and prolong life significantly. Patients not eligible for surgery or SRS are usually offered WBRT alone or combined with chemotherapy depending on the presence of active systemic disease (Morris *et al*, 2004). Those with poor Karnofsky performance score (KPS) may be managed with steroids and other supportive measures. Generally accepted standards for the application of different treatment modalities in patients with brain metastases from melanoma do not exist so far (Douglas and Margolin, 2002; McWilliams *et al*, 2003).

The aim of this retrospective study was to analyse prognostic factors, effects of treatment and survival outcome of relatively non-selected patients with melanoma metastatic to the brain to identify meaningful prognostic groups with respect to overall survival.

## Patients and methods

From October 1986 until March 2003, 265 cutaneous melanoma patients were diagnosed with cerebral metastasis in the University Medical Center of Tuebingen in Southern Germany. Systemic screening and staging algorithms according to valid national surveillance guidelines were used (Orfanos *et al*, 1994; Garbe *et al*, 2006). These algorithms consider the individual risk of relapses for each patient. Prognostic factors include, for example, tumour thickness according to Breslow and the development of previous metastases. Recently, a surveillance programme for stage I–IV patients is used; for example, stage III patients are staged once a year via a computerised tomography (CT)-scan. Stage II patients are followed-up via clinical inspections, lymph node ultrasound and the evaluation of the tumour marker S-100.

Clinical and pathological data on these patients were obtained from the electronic database of the Central Malignant Melanoma Registry, which is a hospital-based registry (Garbe *et al*, 2000; Lasithiotakis *et al*, 2007). In addition, medical records were reviewed to obtain details from operative, pathological and radiation therapy reports. All received data were recorded with standardised forms and finally computerised. The following information was obtained: demographics, anatomical localisation and histological characteristics of primary melanoma, time interval between diagnoses of primary melanoma and brain metastases, site of primary cutaneous melanoma, clinical presentation of brain metastases, localisation of brain metastases, number and size of brain metastases; number and location of other melanoma metastases, KPS, serum lactate dehydrogenase at time of presentation of brain metastases, administered treatment, response to treatment, date of death or last follow-up and cause of death. Diagnosis of brain metastases was mainly based on magnetic resonance imaging (MRI). In a few cases that were diagnosed during the earlier years of the study, CT was used instead. No specific treatment protocol was used. Usually, patients were offered surgery if they had a single or a few surgically accessible brain metastases and a good KPS. If, brain metastases was difficult to access surgically SRS was offered instead. Only 10 out of 94 patients treated with surgery or SRS had more than two brain metastases (eight surgery and two SRS). In these cases, decision was based on clinical experience. Treatment with SRS was delivered using a linear accelerator in all cases. Patients, who received WBRT, were usually treated with a total dose of 30 Gy with a fractionation of 2 Gy (5 days a week for 3 weeks). Tumour response was evaluated using CT and/or MRI every 3 months after therapy. Response to treatment was described using the RECIST criteria (Therasse *et al*, 2000).

### Statistical analysis

Follow-up time was defined as the date of last follow-up or death minus date of diagnosis of brain metastases (overall survival). Brain metastasis diagnosed within 30 days of diagnosis of the primary melanoma was considered synchronous. Survival was calculated from the date of diagnosis of brain metastases. Follow-up time was described as a median value with interquartile range (IQR). Only deaths caused by melanoma were considered in survival analysis. Melanoma-specific survival curves and estimated median survival with relative 95% confidence intervals (95% CIs) were generated according to the Kaplan–Meier product-limit method and were compared by the two-sided log-rank test. Variables proven significant in the univariate analyses at the 0.2 level were included in a Cox proportional hazards model. As radiological response mainly depends on the administered therapy, we excluded the variable ‘response to therapy’ from multivariate analysis to have a better insight to the influence of the indication for treatment on overall survival. Age was entered in the multivariate analysis as continuous variables. Categorical variables were dummy-coded and *P*-values were based on the Wald test. We examined the graphic plot of the log–log survivor function for the variables entered in the analysis. No violation of the proportional hazards assumption was found. Explorative classification and regression tree analysis (CRT) was used to define meaningful prognostic groups with respect to survival probability using information gained from Cox modeling regarding the independent prognostic factors. Minimum size of parental and child nodes were defined as 50 and 20 participants, respectively. Final groups of CRT analysis were presented with median survival time (months) and approximate 95% confidence intervals. Kaplan–Meier curves for the final prognostic groups were generated and compared pairwise by means of log-rank test. Throughout the analysis, two-sided *P*-values <0.05 were considered statistically significant. All statistical analyses were performed using the Statistical Package for Social Sciences (SPSS) version 13.0 (SPSS Inc., Chicago, IL, USA).

## Results

A total of 265 patients with cerebral metastasis because of cutaneous melanoma were included in our analysis. The clinical and pathological characteristics of these patients are presented in Table 1. There were 154 men (58.1%) and 111 women (41.9%). The median age at the time of diagnosis of brain metastasis was 58 years (IQR: 22) and did not differ significantly between men and women (*P*=0.076). Cerebral metastases were present at initial diagnosis of the primary melanoma in 14 patients (5.3%). Eight patients (3.0%) had an unknown primary lesion. In these patients, the diagnosis of melanoma was based on histological examination of excised metastases. Forty-six patients (17.4%) presented with brain metastases without other systemic metastasis. However, a significant proportion of patients (82.3%) suffered from metastatic disease to organs other than the brain. Six patients (2.2%) developed brain metastases directly from stage I, 28 (10.6%) from stage II and 62 (23.4%) from stage III. The rest of the patients were diagnosed with already present stage IV disease.

The most common extracerebral sites of metastasis were lung (62.6%), liver (38.9%) and distant skin/soft tissue (37.4%). Approximately, half of the patients (55.6%) had single or two brain metastases as determined by CT or MRI. Ninety-six patients (36.2%) complained of symptoms associated with brain metastases (i.e., headaches, seizures, palsy) that led to the diagnosis of the disease because of the performance of brain scans. The other 64% were asymptomatic and diagnosed during surveillance.

In all, 128 patients (48.3%) had undergone systemic therapy (chemotherapy or interferon) before the diagnosis of the brain metastases. On the diagnosis of cerebral metastasis a therapy was initiated for 256 (96.6%) patients. Nine patients (3.4%) died before the initiation of any treatment.

We identified 12 different therapy combinations applied (data not shown). Therefore, we created five hierarchical therapy groups in the following order: neurosurgical operation ranked first followed by SRS, WBRT, systemic chemotherapy and finally the group that received no treatment. Patients who underwent neurosurgical resection or SRS were classified in OP or SRS group, respectively, regardless of other therapies administered (i.e., WBRT or chemotherapy). Patients who did not undergo neurosurgical operation or SRS but who underwent WBRT were classified as WBRT regardless of additional chemotherapy. Patients treated with systemic chemotherapy without surgery, SRS or WBRT comprised the ‘systemic chemotherapy’ group. Patients receiving no treatment or only corticosteroids were classified as ‘no therapy’. The distribution of the patients in the treatment groups according to the aforementioned definitions is presented in Table 1. In total, 63 patients (23.8%) underwent neurosurgery (group OP), 31 (11.7%) were treated with SRS, 122 (46.0%) comprised the WBRT group and 22 patients (8.3%) were administered systemic chemotherapy. The remaining 12 patients (4.5%) were classified in the ‘no therapy group’.

During follow-up, 253 (95.5%) of the patients succumbed to their disease. The median overall survival of all 265 patients was 5 months (95% CI: 4.3–5.7). All patients except for one died because of melanoma. Therefore, disease-specific survival should be identical with the overall survival of our cohort. In the univariate survival analysis, age at the time of diagnosis of brain metastases (*P*=0.004), number of brain metastases (*P*<0.001), localisation of brain metastases (supra-, infratentorial) (*P*<0.001), meningeal disease (*P*=0.002), serum lactate dehydrogenase level (*P*<0.001), KPS (*P*<0.001), number of extracerebral metastasis sites (*P*<0.001), presence of liver (*P*<0.001), bone (*P*=0.001), distant lymph node (*P*=0.006), distant skin/soft tissue metastasis (*P*=0.016), classification according to the RTOG classes (Gaspar *et al*, 1997) (*P*<0.001) as well as type of treatment (*P*<0.001) and response to treatment (*P*<0.001) were significantly associated with the overall survival (Table 1). Women had slightly longer overall survival as compared with men but this difference did not reach statistical significance (*P*=0.100). Similarly, diameter of largest brain metastasis larger than 15 mm, presence of symptoms related to brain metastases, presence of adrenal gland, spleen or locoregional metastases were associated with shorter median survival without reaching statistical significance (*P*>0.05 for each variable) (Table 1). Presence of lung metastasis at the time of diagnosis of brain metastases (*P*=0.121), as well as the stage of primary cutaneous melanoma (*P*=0.5), were not significantly associated with the overall survival. There were also no statistically significant survival differences between the 5-year periods of diagnosis of brain metastases: 1986–1990: 3.2 months (95% CI 2.8–7.6), 1991–1995: 5.6 months (95% CI 3.5–7.3), 1996–2000: 5.4 months (95% CI 4.2–7.3), 2001–2005: 5.6 months (95% CI 4.7–7.5), (*P*=0.262).

**Figure 1:**
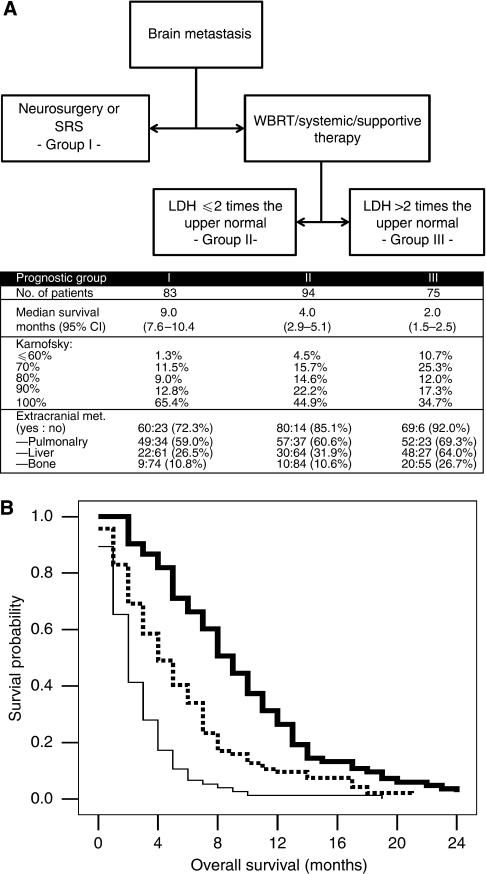
(**A**) Classification and regression tree analysis. Prognostic groups were defined by means of the defining variables and the respective median survival with its respective 95% confidence intervals. SRS: stereotactic radiosurgery, WBRT: whole brain radiation therapy, LDH(s): serum levels of lactate dehydrogenase. (**B**) Overall survival of patients with brain metastasis from cutaneous melanoma by prognostic group (group I: bold line, group II: broken line, group III: thin line). Overall and pairwise, the three final groups were significantly different with respect to survival (log rank *P*<0.001, group I *vs* II; *P*<0.001, group I *vs* III; *P*<0.001, group II *vs* III).

A stepwise Cox multivariate analysis was performed to determine the independent predictors of the overall survival. The results of the multivariate analysis are shown in Table 2. The independent prognostic factors of the overall survival were: serum lactate dehydrogenase (*P*<0.001), administered therapy (*P*=0.002), number of brain metastases (*P*=0.032) and presence of bone metastasis (*P*=0.044). CRT analysis identified three final prognostic groups (Figure 1A). Group I comprised patients treated with neurosurgery or stereotactic surgery. In group II were included patients treated with WBRT or chemotherapy or supportive therapy who had serum levels of serum lactate dehydrogenase equal or less than two times the maximum normal value. In group III were classified patients treated with WBRT or chemotherapy or supportive therapy who had serum lactate dehydrogenase more than two times the maximum normal value. For each of these groups Kaplan–Meier survival curves were computed and compared by means of log-rank test (Figure 1B). Overall and pairwise, the three final groups were significantly different with respect to survival (overall: *P*<0.001, group I *vs* II: *P*<0.001, group I *vs* III *P*<0.001, group II *vs* group III: *P*<0.001). Survival rates for each subgroup are shown in Figure 1. Group I was the group with the most favourable prognosis with median survival of 9.0 months, followed by group II (median survival 4.0 months) and group III (median survival 2.0 months). Comparing the results of the recursive partition analysis (RPA) of the Radiation Therapy Oncology Group (RTOG), and our CRT analysis revealed significant different survival rates for both methods. However, although the RPA allocates most of the patients into class II, the CRT differs the patients into the three prognostic groups more balanced (Table 3).

## Discussion

In this retrospective analysis, we aimed to identify determinants of the outcome of patients with brain metastasis from cutaneous melanoma. The overall survival of our patients was generally very poor with median survival time not exceeding 5 months. Comparable overall survival times have been shown by a few other studies (Buchsbaum *et al*, 2002; Harrison *et al*, 2003; Fife *et al*, 2004).

The results of our multivariate Cox analysis showed that the main independent favourable prognostic factors in these patients are the levels of serum lactate dehydrogenase (normal *vs* elevated), the type of administered therapy (neurosurgery or SRS *vs* other), the number of brain metastases (single *vs* multiple) and the presence of bone metastasis (false *vs* true). By means of explorative classification analysis, we identified three different prognostic groups with statistically significant differences in the overall survival. Patients treated with either neurosurgery or SRS comprised the group with the most favourable prognosis. The rest of the patients could be further sub-classified into two groups based on the levels of serum lactate dehydrogenase (⩽2 upper normal value *vs* >2 upper normal value).

This is the first study to identify different prognostic groups by means of classification trees analysis in a relatively unselected population of patients with brain metastases from melanoma particularly by taking into account the levels of serum lactate dehydrogenase, which is a well-established prognostic factor in stage IV melanoma (Manola *et al*, 2000; Balch *et al*, 2001). Normally serum lactate dehydrogenase is rising with increasing tumour masses and can therefore be used as a surrogate marker for tumour load (Agarwala *et al*, 2009). The use of recursive partitioning analysis grouping, as described by the RTOG, has been suggested as a tool for the stratification of patients with brain metastases (Gaspar *et al*, 1997). However, this system does not take into account particularities of different tumour types (like serum lactate dehydrogenase levels for melanoma) and for this reason we performed detailed analysis to identify more meaningful tools for classification of patients according to their prognosis in our population.

Our findings are in keeping with those of Sirott *et al* who showed that in stage IV melanoma patients pre-treatment serum lactate dehydrogenase level is associated with the overall survival with a continuous rather than a discrete pattern. Increasing levels of serum lactate dehydrogenase were associated with shorter survival and the optimal cut-off point to identify different prognostic groups was serum level of serum lactate dehydrogenase exceeding double of upper normal value (Sirott *et al*, 1993). Taking into account the short period of median survival of 2 months for patients in group III we suggest being restrictive in applying aggressive chemotherapy or WBRT because these patients seem not to benefit.

Patients with brain metastasis usually receive multimodality treatment including surgery, radiation therapy, chemotherapy, and so on. In our sample, we identified 12 different therapy combinations. To enable meaningful comparisons we created five hierarchical groups of therapy. This classification was based mainly on that conventional surgery and SRS were mutually exclusive treatment modalities in our sample (data not shown), which are related with significantly longer overall survival compared with single treatment with WBRT, whereas systemic therapy has not been shown to increase overall survival in patients with brain metastasis from melanoma (Sampson *et al*, 1998; Wronski and Arbit, 2000; Douglas and Margolin, 2002). The overall survival did not differ significantly between patients treated with neurosurgery or SRS, which is in line with the findings from recent retrospective and one randomised study (Fife *et al*, 2004; Aoyama *et al*, 2006; DiLuna *et al*, 2007). However, the indications between direct surgery and SRS differ significantly and depend mainly on the size, the number and the anatomical site of metastatic lesions. These factors have a major effect on overall survival of patients with brain metastases and until now there is no randomised study between the two modes. As morbidity related with neurosurgery, is much higher compared with SRS, it would be of great importance to identify subgroups of patients who might benefit equally from SRS compared with conventional surgery. This could be ideally investigated in the frame of randomised studies.

In our sample, more than half of the patients had less than three brain metastases with 40% having single brain metastasis. Similar results have been reported by Sampson *et al* (1998). Fine *et al* reported a much lower rate of single brain metastasis (23%) (Fife *et al*, 2004). However, in their study the rate of unknown cases was 50% that makes a valid comparison difficult. In addition, the majority of our patients were asymptomatic and diagnosed by brain scans performed during regular surveillance according to national German guidelines (Orfanos *et al*, 1994; Garbe *et al*, 2006). This approach is different compared with other countries such as the United Kingdom or the United States where scans are only performed in case of cerebral symptoms.

We found that a significant number of patients (17%) presented without evidence of extracranial disease, whereas another 17% had single organ extracranial involvement. These rates are much lower compared with those reported by Sampson *et al* that might be explained by selection of patients with more favourable prognostic features to be referred to a neurosurgical department or by underestimation of the true number of metastasis by early generation CT or MRI as it was postulated by the investigators (Sampson *et al*, 1998). In any case, a significant proportion of patients with brain metastasis from melanoma, exceeding one-third of the total in our sample, could be considered candidates for local treatment with neurosurgery or SRS. The survival rates of these patients are comparable with those reported for patients with stage IV melanoma (Balch *et al*, 2001). This is a very interesting finding, which argues against the general exclusion of patients with brain metastases from large clinical studies in melanoma, particularly in view of current evidence suggesting that chemotherapy, which is active against extracranial metastasis may also be effective against brain metastasis (Kaufmann *et al*, 2005; Gerstner and Fine, 2007; Ranson *et al*, 2007).

In studies analysing medical outcomes over a long period of time, data heterogeneity are always a concern. Improvement in surgery and oncotherapy during a 17 years period might influence the results of treatment of brain metastasis from melanoma. However, when we analysed survival of sequential 5-year periods no significant difference was evident. Our retrospective analysis neither aimed nor had the strength to evaluate different therapeutic modalities in the field of brain metastasis in melanoma. This has to be investigated by means of targeted prospective studies. Moreover, we did not analyse data regarding administered therapies before diagnosis of brain metastasis and we did not have information regarding specific complications of the disease, which led to death. This would be of interest because extracranial disease represents a major limitation on survival in patients receiving focal treatment for melanoma brain metastasis (Hasegawa *et al*, 2003). We did not have information on the number of SRS procedures received by the patients, which might have an effect in the overall survival as it is recently suggested by a retrospective study (Samlowski *et al*, 2007). In contrast, we had detailed data with negligible rates of missing cases for most of the variables we analysed and almost complete follow-up data for the entire of our collective.

In conclusion, the prognosis of patients with brain metastasis from cutaneous melanoma is ominous necessitating the development of effective focal and systemic therapies. The independent prognostic factors of these patients were the level of serum lactate dehydrogenase, the administered therapy, the number of brain metastases and the presence of bone metastasis. On the basis of the administered therapy and the levels of serum lactate dehydrogenase, three different prognostic groups with statistically different overall survival could be identified. Patients treated with conventional neurosurgery or SRS comprised the group with the highest overall survival, which was comparable with the overall survival of general stage IV melanoma patients; a finding arguing against the exclusion of such patients from clinical trials solely on the basis of dismal prognosis.

## Figures and Tables

**Table 1:** Patients and brain metastasis characteristics

	**Patients *N* (%)**	**Median survival months (95% CI)**	***P*** a
*Gender*			
Male	154 (58.1)	5.0 (4.0–6.0)	**0.100**
Female	111 (41.9)	5.0 (3.8–6.2)	
			
*Age* b			
<45years	68 (25.7)	7.0 (5.4–8.6)	**0.004**
>44years	197 (74.3)	4.0 (3.2–4.8)	
			
*AJCC stage of primary melanoma*			
I	66 (24.9)	5.0 (4.0–6.0)	**0.500**
II	82 (30.9)	5.0 (4.0–6.0)	
III	67 (25.3)	4.0 (3.4–4.6)	
IV	31 (11.7)	5.0 (3.0–7.0)	
Unknown	19 (7.2)		
			
*Distant disease free interval*			
<24 months	133 (50.2)	4.0 (3.1–4.9)	**0.003**
⩾24 months	132 (49.8)	6.0 (5.0–7.0)	
			
*Brain metastasis free interval*			
<24 months	113 (42.6)	4.0 (3.1–4.9)	**0.015**
⩾24 months	152 (57.4)	6.0 (5.0–7.0)	
			
*Symptomatic brain metastasis* b			
Yes	96 (36.2)	4.0 (2.5–5.5)	**0.120**
No	147 (55.5)	5.0 (4.1–5.9)	
Unknown	22 (8.3)		
			
*Number of brain metastases* b			
1	107 (40.4)	8.0 (6.3–9.7)	**<0.001**
2	40 (15.1)	5.0 (3.7–6.3)	
Multiple	118 (44.5)	3.0 (2.2–3.8)	
			
*Localization of brain metastases* b			
Supratentorial	191 (72.1)	6.0 (5.2–6.9)	**<0.001**
Infratentorial	16 (6.0)	4.0 (4.2–5.8)	
Both	43 (16.2)	2.0 (1.2–2.8)	
Unknown	15 (5.7)		
			
*Meningeal disease*			
Yes	6 (2.3)	2.0 (1.4–2.6)	**0.002**
No	259 (97.7)	5.0 (4.2–9.0)	
			
*Diameter of largest brain metastases* b			
⩽15 mm	124 (46.8)	6.0 (4.5–7.5)	**0.088**
>15 mm	91 (34.3)	5.0 (4.2–5.8)	
Unknown	50 (18.9)		
			
*LDH* b			
Normal range	100 (37.7)	7.0 (6.0–8.1)	**<0.001**
× 1–2 times maximum normal values	72 (27.2)	3.0 (2.2–3.8)	
> × 2 times maximum normal values	23 (8.7)	1.0 (0.3–1.7)	
Unknown	70 (26.4)		
			
*Karnofsky performance score* b			
>80%	168 (63.4)	6.0 (5.0–7.0)	**<0.001**
⩽80%	86 (32.4)	3.0 (2.4–3.6)	
Unknown	11 (4.2)		
			
*Number of extracerebral metastasis sites* b			
0	46 (17.4)	7.0 (5.1–8.9)	**<0.001**
1	45 (17.0)	7.0 (5.2–8.9)	
2	62 (23.4)	4.0 (2.9–5.1)	
3	48 (18.1)	3.0 (1.1–4.9)	
⩾4	64 (24.2)	3.0 (2.0–4.0)	
			
*Presence of locoregional metastasis* b			
Yes	178 (67.2)	4.0 (3.1–4.9)	**0.156**
No	87 (32.8)	6.0 (5.1–7.0)	
			
*Presence of distant lymph node metastasis* b			
Yes	96 (36.2)	3.0 (2.0–4.0)	**0.006**
No	169 (63.8)	5.0 (4.1–6.0)	
			
*Presence of distant skin/soft tissue metastasis* b			
Yes	99 (37.4)	3.0 (2.0–4.0)	**0.016**
No	166 (62.6)	5.0 (4.2–5.8)	
			
*Presence of lung metastasis* b			
Yes	166 (62.6)	5.0 (4.2–5.8)	**0.121**
No	99 (37.4)	5.0 (3.5–6.5)	
			
*Presence of liver metastasis* b			
Yes	103 (38.9)	3.0 (2.0–4.0)	**<0.001**
No	162 (61.1)	6.0 (5.1–6.9)	
			
*Presence of bone metastasis* b			
Yes	41 (15.5)	3.0 (2.2–3.8)	**0.001**
No	224 (84.5)	5.0 (4.1–5.9)	
			
*Presence of adrenal gland metastasis* b			
Yes	32 (12.1)	4.0 (1.3–6.7)	**0.126**
No	233 (83.9)	5.0 (4.2–5.8)	
			
*Presence of spleen metastasis* b			
Yes	29 (10.9)	4.0 (1.4–6.6)	**0.400**
No	236 (89.1)	5.0 (4.1–5.9)	
			
*RTOG Classes*			
Class I	29 (11.9)	9.0 (7.0–15.0)	**<0.001**
Class II	180 (67.9)	5.0 (4.0–6.0)	
Class III	56 (21.3)	2.0 (2.0–3.0)	
			
*Administered therapy*			
Neurosurgical operation	63 (24.6)	9.0 (7.0–11.0)	**<0.001**
SRS	31 (12.1)	9.0 (7.0–11.1)	
WBRT	122 (47.7)	4.0 (3.2–4.8)	
Chemotherapy	28 (10.9)	3.0 (1.6–4.4)	
No therapy	12 (4.7)	1.0 (0.1–1.9)	
			
Neurosurgical operation or SRS	94 (36.7)	9.0 (8.0–11.0)	**<0.001**
WBRT	122 (47.7)	4.0 (3.2–4.8)	

Abbreviations: AJCC=American Joint Commission on Cancer; BM=brain metastasis; LDH=serum lactate dehydrogenase; RTOG=Radiation Therapy Oncology Group; SRS=stereotactic radiosurgery; WBRT=whole brain radiation therapy.

a Log-rank test.

b At the time of diagnosis of the brain metastasis.

**Table 2:** Multivariate analysis of prognostic factors

	**HR (95%CI)**	***P*** a
*Number of brain metastases*		
1 brain metastasis	Ref	**0.032**
2 brain metastases	1.5 (0.9–2.5)	
Multiple brain metastases	1.9 (1.2–3.2)	
		
*LDH*		
Normal range	Ref	**<0.001**
⩽ × 2 max normal	2.7 (1.8–4.0)	
> × 2 max normal	7.3 (3.7–14.3)	
		
*Presence of bone metastasis*		
No	Ref	**0.044**
Yes	1.7 (1.0–2.8)	
		
*Administered therapy*		
Surgery	Ref	**0.002**
SRS	1.0 (0.6–1.8)	
WBRT	1.5 (0.9–2.5)	
Chemotherapy	1.8 (0.9–3.4)	
No therapy	5.4 (2.3–12.6)	

Abbreviations: BM=brain metastasis; CI=confidence interval; HR=hazard ratio; SRS=stereotactic radiosurgery; WBRT=whole brain radiation therapy.

a Significance based on Wald test.

Hazard ratio with its 95% confidence interval.

**Table 3:** Allocation of the patients into different prognostic groups according to recursive partition analysis of the RTOG and CRT analysis

	**CRT I (%)**	**CRT II (%)**	**CRT III (%)**
RTOG I	7.9	2.8	0.4
RTOG II	21.4	27.0	18.7
RTOG III	3.6	7.5	10.7

Abbreviations: CRT= classification and regression tree; RTOG=Radiation Therapy Oncology Group.
